# Comparative genomics of *Beauveria bassiana*: uncovering signatures of virulence against mosquitoes

**DOI:** 10.1186/s12864-016-3339-1

**Published:** 2016-12-01

**Authors:** Claudio A. Valero-Jiménez, Luigi Faino, Daphne Spring in’t Veld, Sandra Smit, Bas J. Zwaan, Jan A. L. van Kan

**Affiliations:** 1 Laboratory of Genetics, Wageningen University, Droevendaalsesteeg 1, 6708 PB Wageningen, The Netherlands; 2 Laboratory of Entomology, Wageningen University, Droevendaalsesteeg 1, 6708 PB Wageningen, The Netherlands; 3 Laboratory of Phytopathology, Wageningen University, Droevendaalsesteeg 1, 6708 PB Wageningen, The Netherlands; 4 Bioinformatics Group, Wageningen University, Droevendaalsesteeg 1, 6708 PB Wageningen, The Netherlands

**Keywords:** *Beauveria bassiana*, Comparative genomics, Virulence, Genome sequencing

## Abstract

**Background:**

Entomopathogenic fungi such as *Beauveria bassiana* are promising biological agents for control of malaria mosquitoes. Indeed, infection with *B. bassiana* reduces the lifespan of mosquitoes in the laboratory and in the field. Natural isolates of *B. bassiana* show up to 10-fold differences in virulence between the most and the least virulent isolate. In this study, we sequenced the genomes of five isolates representing the extremes of low/high virulence and three RNA libraries, and applied a genome comparison approach to uncover genetic mechanisms underpinning virulence.

**Results:**

A high-quality, near-complete genome assembly was achieved for the highly virulent isolate Bb8028, which was compared to the assemblies of the four other isolates. Whole genome analysis showed a high level of genetic diversity between the five isolates (2.85–16.8 SNPs/kb), which grouped into two distinct phylogenetic clusters. Mating type gene analysis revealed the presence of either the *MAT*1–1–1 or the *MAT*1–2–1 gene. Moreover, a putative new *MAT* gene (*MAT*1-2–8) was detected in the *MAT*1–2 locus. Comparative genome analysis revealed that Bb8028 contains 163 genes exclusive for this isolate. These unique genes have a tendency to cluster in the genome and to be often located near the telomeres. Among the genes unique to Bb8028 are a Non-Ribosomal Peptide Synthetase (NRPS) secondary metabolite gene cluster, a polyketide synthase (PKS) gene, and five genes with homology to bacterial toxins. A survey of candidate virulence genes for *B. bassiana* is presented.

**Conclusions:**

Our results indicate several genes and molecular processes that may underpin virulence towards mosquitoes. Thus, the genome sequences of five isolates of *B. bassiana* provide a better understanding of the natural variation in virulence and will offer a major resource for future research on this important biological control agent.

**Electronic supplementary material:**

The online version of this article (doi:10.1186/s12864-016-3339-1) contains supplementary material, which is available to authorized users.

## Background

*Beauveria bassiana* is a cosmopolitan entomopathogenic fungus (EPF) with a broad host range covering most insect orders (e.g., Coleoptera, Diptera, Hemiptera, Lepidoptera) and also arthropods (subclass Acari) [1, 2]. *B. bassiana* is currently used commercially as a biological control agent for different arthropod pests in China such as the pine caterpillar (*Thaumetopoea pityocampa)*, corn borer (*Ostrinia furnacalis*), peach aphid (*Myzus persicae*), and pine sawyer (*Bursaphelenchus xylophilus*) [3]. *B. bassiana* is also being developed as a biological control tool for vectors of human diseases, such as *Anopheles* mosquitoes that transmit malaria [4, 5], *Aedes aegypti* mosquitoes that are vectors of Dengue, Chikungunya and Zika virus [6, 7], the *Triatoma infestans* bug, the Chagas disease vector [8], and *Ixodes ricinus* ticks that transmit Lyme disease [9].

Infection by *B. bassiana* starts when conidia adhere to the host cuticle via hydrophobic interactions and subsequently germinate. A germ tube is formed that is able to penetrate the cuticle through a combination of mechanical pressure and cuticle degrading enzymes. Once the fungal structures reach the haemocoel, the fungus switches to growth as single-celled blastospores, which are capable of evading the insect immune system, exploit the haemolymph for nutrition, and secrete toxins that eventually kill the host. Once the host is dead, the fungus breaches the cuticle from the inside outwards, allowing the formation of conidia that upon dispersal start new infections [10].

A good understanding of the life history of *B. bassiana* as well as the genetic mechanisms of virulence is crucial to develop it as an effective and sustainable biological control agent. Substantial progress has been made in the genetic characterization of the mechanisms involved in the infection cycle of *B. bassiana* inside insects. Many of the recent findings regarding genes involved in virulence were possible due to the development of reliable protocols that allow genetic manipulation [11] together with the availability of a draft genome sequence [12]. For instance, it has been found that *B. bassiana* expresses two hydrophobin genes, *hyd1* and *hyd2*, that are believed to be involved in surface hydrophobicity, adhesion, virulence and composition of the rodlet layer. A *Δhyd1* knock-out mutant showed decreased virulence and spore hydrophobicity but unchanged surface adhesion, while a *Δhyd2* mutant showed reduced hydrophobicity and surface adhesion but unchanged virulence [13]. Similarly, a cytochrome P450 gene, *Bbcyp52x1*, was shown to function in enzymatic breakdown of lipids in the waxy layer. A *ΔBbcyp52x1* knock-out mutant showed reduced virulence on *Galleria mellonella* upon topical application of conidia, though not upon intra-hemocoel injection [14].

Despite all efforts made to understand virulence in *B. bassiana* (for a review, see [15]), many of the genes that were functionally analysed thus far are involved in general biological processes (e.g., conidiation, stress response) that pleiotropically affect virulence. This indicates that virulence is tightly connected to the biology and life history of *Beauveria*. In order to increase the efficacy and specificity of *Beauveria* against arthropods, we need to identify the genes exclusively implicated in entomopathogenic virulence. Therefore, strategies must be employed that allow *de novo* identification of genes and mechanisms specifically related to virulence. Our previous identification of ample natural genetic variation for virulence in *B. bassiana* isolates [16] opens the way for a comparative genomic approach. Such an approach offers a powerful opportunity to collect comprehensive information of genetic variation and identification of variants of virulence and their evolutionary dynamics. Importantly, knock-out mutant approaches are crucial and will play an important role in verifying the action of the candidate genes that emerge from comparative genomics of natural isolates that differ in virulence.

The aim of this study was to improve our knowledge in understanding the key factors involved in virulence of *B. bassiana* by a comparative genomics study. *B. bassiana* is well known for having a broad genetic, host, and virulence diversity. As a consequence, genome structure and DNA sequence variation between any two isolates could be related to many factors in the biology of this fungus (including neutral evolution/genetic drift). Therefore, we sequenced five isolates of contrasting virulence, selected from a panel of 29 *B. bassiana* isolates of which the natural variation in virulence had been previously characterized [16]. We assembled a gapless near-complete genome for the most virulent *B. bassiana* isolate, Bb8028, which provides the community a highly suitable reference genome to be used for comparison with other isolates.

## Methods

### Fungal isolates and maintenance

*Beauveria bassiana* isolates ARSEF 1520 (Bb1520), ARSEF 2597 (Bb2597), ARSEF 4305 (Bb4305), ARSEF 5078 (Bb5078) and ARSEF 8028 (Bb8028) were obtained from the USDA-ARS Collection of Entomopathogenic Fungal Cultures (ARSEF). These isolates were selected based on their previously characterized virulence against malaria mosquitoes. Isolates Bb2597 and Bb8028 were previously shown to display high virulence, Bb1520 has intermediate virulence, while Bb4305 and Bb5078 have low virulence [16]. Isolates were grown on Sabouraud Dextrose Agar with 1% yeast extract (SDAY) at 27 °C before DNA extraction and sequencing.

### Genome and transcriptome sequencing

DNA was isolated from freeze-dried mycelium using a CTAB-based method [17]. All isolates were sequenced with paired-end libraries of 180 bp insert size, and with a read length of 2x100 bp. Isolates Bb1520, Bb2597, and Bb8028 were further sequenced with a mate-pair library of 10 kb (Bb1520, Bb2597) or 6 kb (Bb8028). Isolate Bb8028 was selected as a reference based on the quality of the assembly, therefore additional sequencing focussed on this isolate and was performed using two PacBio SMRT cells with an insert size of 20 kb. The paired-end and mate-pair sequencing were performed using Illumina HiSeq2000 technology at the Beijing Genome Institute (BGI, Hongkong, China). PacBio sequencing was performed using the RS II instrument at Keygene N.V. (Wageningen, the Netherlands).

To obtain the transcriptome, RNA from Bb8028 was isolated from three different sources: (1) 5-day old mycelia grown on rice medium; (2) proto fruiting bodies (stromata like-structures) obtained from a cross between Bb8028 and Bb5078 co-cultivated for 2 months on rice medium as described [18]; and (3) infected mosquitoes that had just died. Rice medium was composed of 60 g of brown rice and 10 g of dried silkworm pupae, and 100 ml of distilled water. RNA was isolated using a RNAeasy Plant mini Kit (Qiagen) following manufacturer’s recommendations and sequenced using a paired-end library of 180 bp insert size on an Illumina HiSeq2000 platform at BGI.

### Genome assembly and annotation

For isolates Bb4305 and Bb5078, *de novo* assembly was performed using Celera assembler [19] with default settings except k-mer size, which was set to 15. Isolates Bb1520, Bb2597, and Bb8028 were assembled using Allpaths-LG [20] with default settings. Additionally, PacBio data was used to improve the assembly of Bb8028 using PBJelly [21] and quiver tools [22], and erroneously merged contigs were manually corrected. RNA-Seq libraries were assembled *de novo* with Oases [23] and Trinity [24]. Also, the RNA-Seq reads were mapped to the Bb8028 assembly using Tophat [25]. Completeness of the genome assemblies was assessed by the Benchmarking Universal Single-Copy Orthologs (BUSCO) v1.1b1 software tool [26]. RepeatMasker [27] was used to search for homologs of known repeats using the library from Repbase [28], and previously unknown repeats were predicted *de novo* with RepeatScout [29]. Local Collinear Blocks (LCBs) were identifed using progressiveMauve (build date Feb 25, 2015), with default settings. Parsing and inversion detection was performed using a custom python script. Genome annotation was performed using the MAKER (v.2.31.8) pipeline [30] with AUGUSTUS [31], SNAP [32], and GeneMark-ES [33] as ab initio gene predictors. The assembled RNA-Seq reads and all fungal proteins available in the Uniprot database were used as evidence for gene prediction. Furthermore, the predicted proteins were manually inspected and curated. The predicted proteins were functionally annotated using BLASTp [34] against the non-redundant database of the National Center of Biotechnology Information (NCBI) and InterProScan [35]. The BLASTp and InterproScan results were imported into Blast2GO version 3.2 [36] and Gene Ontology (GO) terms were predicted. Overrepresentation of GO terms was analysed using Fisher’s test as embedded in Blast2GO. Secreted proteins were predicted by SignalP 4.1 [37].

### Pan-genome analysis

The pan-genomes and core genomes at the gene level were estimated using OrthoMCL [38] implemented in GET_HOMOLOGUES-EST [39] with e-value 1e^−5^ and 75% coverage. Besides the five newly sequenced genomes presented here, the previously reported genome of *B. bassiana* ARSEF 2860 (Bb2860) [12] was also included in the analysis.

### Phylogenetic analysis

The phylogenetic relationships were determined with the software REALPHY [40] between all isolates of *B. bassiana*, including the previously sequenced isolate Bb2860 [12], and using *C. militaris* [41] as the outgroup of the tree. All genomes were mapped using Bowtie2 [42] on the genome of Bb8028 as a reference. After the alignment, a maximum likelihood phylogenetic tree was inferred with RAxML [43] using the generalised time reversible (GTR) plus GAMMA nucleotide substitution model and 100 rapid bootstraps.

### Variant analysis

To identify single-nucleotide polymorphisms (SNPs), Freebayes (v0.9.21), a Bayesian method based program, was used [44] with a minimum alteration fraction of 0.90. All sequences were mapped with BWA [45] to the genome of isolate Bb8028. To minimize false positives, SNPs called at positions with low coverage (<10x) were not considered. SNP density and transition/transversion ratios were calculated using VCFtools [46].

### Secondary metabolite analysis

Putative gene clusters that are predicted to be involved in biosynthesis of secondary metabolites were identified using antiSMASH (antibiotics and Secondary Metabolite Analysis SHell) version 3.0.4 [47].

### Mating type gene analysis

The presence of mating type loci in all isolates of *B. bassiana* (including Bb2860) were identified by homology to characterized MAT genes of other filamentous fungi using BLASTp. The mating type alleles *MAT*1–1–1 and *MAT*1–2–1 were also determined by PCR with the primer pairs MAT112.F4/ MAT111.R5 and MAT2.F4/ MAT2.R5 [48], respectively.

### Selection analysis

To detect regions under positive selection, protein-coding DNA alignments based on the orthologous genes were generated with ParaAT. Ratios of non-synonymous (Ka) to synonymous (Ks) nucleotide substitution rates per site were estimated for each alignment using KaKs_Calculator 2.0 [49]. A sliding window of 100 bp was used with a gamma distribution (gMYN) model.

## Results

### A high-quality *Beauveria bassiana* reference genome

Five *B. bassiana* isolates were sequenced that were previously phenotyped for virulence against malaria mosquitoes. Isolate Bb8028, which is consistently the most virulent isolate in our collection [16] was selected to serve as a high quality reference genome for the dataset. This isolate was sequenced with both short and long-reads, single molecule sequencing technology, while two isolates were sequenced with short reads on both short and long insert libraries (Bb1520 and Bb2597), and two isolates were sequenced with short reads only on short insert libraries (Bb4305 and Bb5078). The final assembly sizes were in the range of 34.45–38.83 Mb, slightly larger (33.7 Mb) than the assembly of the previously sequenced isolate of *B. bassiana*, Bb2860 [12]. The final assemblies resulted in 12 to 3,952 contigs (Table 1), and the five isolates were predicted to encode between 10,210 and 10,831 protein-coding genes. The genome assembly of isolate Bb8028 had only 12 contigs with an N50 contig length of 5.69 Mb, and there were no gaps present. The 12 contigs covering the genome of Bb8028 represent four full chromosomes (with telomeric repeats at both ends) and 5 chromosome ends with a single telomeric repeat.

**Table 1:** Summary of the genomic features of the different *B. bassiana* isolates

Genome features	Bb1520	Bb2597	Bb4305	Bb5078	Bb8028
Total Assembly Size (Mb)	36.97	38.83	34.77	34.45	35.02
Sequencing libraries used	PE^a^ + MP^b^	PE + MP	PE	PE	PE + MP + PB^c^
Scaffold No.	251	351	1,694	3,365	-
Scaffold N50 (kb)	1,560	970	58	31	-
Contig No.	1,218	1,356	2,252	3,952	12
Contig N50 (kb)	85	69	35	22	5,685
GC content (%)	49.64	49.11	49.56	51.06	48.66
Protein coding genes	10,599	10,753	10,513	10,831	10,210
Predicted secreted proteins	1097	1063	1087	1069	1075

^a^PE = paired end sequencing of short insert Illumina library

^b^MP = mate pair sequencing of large insert Illumina library

^c^PB = single molecule real-time sequencing of large fragments (PacBio)

The three highest quality assemblies (Bb8028, Bb1520 and Bb2597) were used to explore structural rearrangements between these three genomes. In total, 695 locally collinear blocks (LCBs) were shared between two or three genomes. We identified 18 cases of clear inversions, where three adjacent LCBs were shared between the three genomes and in one genome the middle block was inverted with respect to the others. Fifteen of these inversions occurred in strain Bb1520 (Bb8028 and Bb2597 remain collinear), while two inversions occurred in Bb8028 and one in Bb2597. The size of inverted regions ranged from ~1 kb to 220 kb. Some LCBs demonstrated significant size differences between the genomes (ranging from 3 to 65 kb), suggesting insertions or deletions. Two inversions in isolate Bb1520 occurred within the coding sequence of a predicted gene (in the Bb8028 reference genome), thereby truncating the coding sequence of that gene in Bb1520. The vast majority of inversions occurred outside coding sequences and were judged to have no impact on gene structure or regulatory regions.

**Fig. 1:**
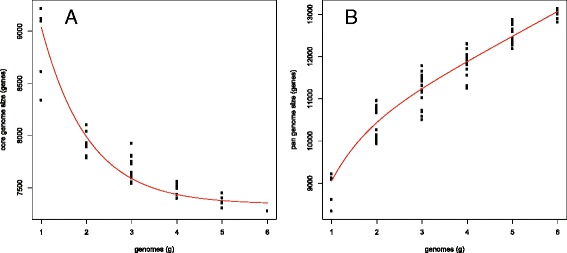
Pan-genome analysis of *B. bassiana* isolates. **a** Estimation of the *B. bassiana* core genome, in which the number of shared genes is plotted as a function of the number of isolates sequentially added. **b** Estimation of *B. bassiana* pan-genome size, in which the numbers of all genes are plotted as a function of the number of isolates sequentially added

The completeness of all five genomes was assessed by checking for the representation of a set of universal single-copy orthologs for fungi [26], and this analysis indicated that each genome had at least 96% of the total BUSCOs (Additional File 1), suggesting a high quality of all the assemblies. The pan-genome is here defined as the combination of all the genes that are present in the genomes of all isolates of a particular species, and it includes the core genes, dispensable genes, and isolate-specific genes [50, 51]. A pan-genome analysis for *B. bassiana* for the five isolates sequenced in this study in combination with the previously sequenced isolate Bb2860 [12], indicated that the core genome of *B. bassiana* consists of 7,341 orthologous gene clusters (Fig. 1a). On the other hand, the pan-genome consists of 13,068 orthologous gene clusters (Fig. 1b).

### High levels of genetic diversity as revealed by SNPs and phylogenetic analysis

**Fig. 2:**
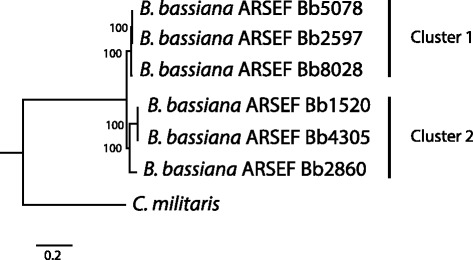
Phylogenetic tree based on whole genome assemblies of the different isolates of *B. bassiana*, with *C. militaris* as the outgroup to root the tree. On the right, the different clusters found are highlighted

The phylogenetic relationships between isolates of *B. bassiana* were determined using the whole genome. Two distinct clusters were formed (Fig. 2), designated 1 and 2. Cluster 1 contains three isolates (Bb2597, Bb5078 and Bb8028), while the other three isolates (Bb1520, Bb4305 and Bb2860) are grouped in cluster 2. The grouping did not reflect a geographical distribution: the two isolates originating from USA were assigned to different clusters, as well as the two isolates of European origin. In addition, the two clusters did not reflect distinct virulence levels, as the low virulence isolate (Bb5078) grouped with the two high virulence isolates in cluster 1.

All the read data that were available in this study were mapped to Bb8028 as a reference in order to detect SNPs. Within cluster 1, there were 2.85 and 4.8 SNPs per kb for isolates Bb5078 and Bb2597, respectively. In contrast, the genetic variation in isolates from cluster 2 against Bb8028 was four-fold higher, with an average of 16.6 and 16.8 SNPs per kb, for Bb1520 and Bb4305, respectively. Similarly, the genetic identity to respect to Bb8028 was higher in Bb5078 (90.4%) and Bb2597 (89.7%), and lower in Bb4305 (88.4%) and Bb1520 (87.9%). We searched for SNPs in coding sequences of a set of 54 genes that have been functionally analysed by knock-out mutagenesis (reviewed in [15]) between the five *B. bassiana* isolates sequenced in this study. There are two genes that contain a SNP with potentially high impact: in the α-glucoside transporter gene *Bbagt*1 in a single isolate, and in the thioredoxin gene *trx*3 in two isolates. The remaining 52 genes do not display any high impact SNPs.

### A new mating type gene

**Fig. 3:**
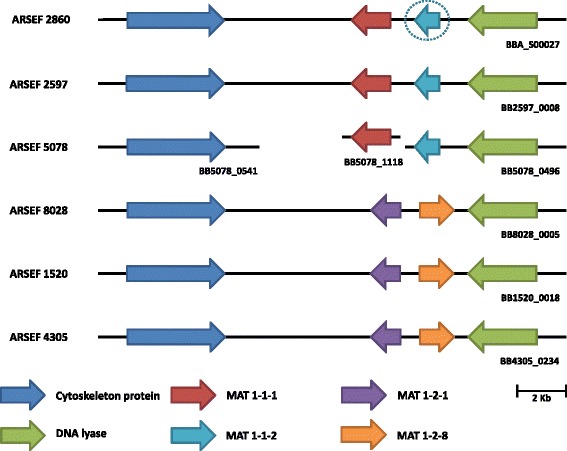
Graphical presentation of the synteny of mating type genes and their flanking region in *B. bassiana* genomes. Genes in the same colour show orthologous relationships. The dashed circle represents a gene that has not previously been identified in the genome of ARSEF 2860

Analysis of orthologous relationships between the genes of all isolates of *B. bassiana* in this study identified the presence of several mating type loci (Fig. 3). We identified two *MAT* genes, *MAT1–1–1* and *MAT1–1–2*, in Bb2597 and Bb5078. The *MAT1–1–2* gene was not previously identified in the published genome of Bb2860 [12], but based on BLAST hits, genes from Bb2597 and Bb5078 have homology to the *MAT1–1–2* gene in *C. militaris* [41]. Further alignment of the putative *MAT1–1–2* gene from Bb2597 and Bb5078 to scaffold BBA_S00027 from Bb2860 confirmed that this gene is also present in Bb2860 but was not characterized [12]. The *MAT*1-2 locus was identified by homology to the HMG-box domain in Bb1520, Bb4305 and Bb8028. A putative new *MAT* gene was found next to the *MAT*1–2–1 gene. BLASTp analysis and sequence alignments shows that it has partial homology to the newly described *MAT*1–2–8 gene in *Villosiclava virens* [52]. PCR confirmed presence of either *MAT*1–1–1 or *MAT*1–2–1 in each isolate (Additional File 2).

The draft genome of *B. bassiana* isolate Bb2860 was reported to lack the *spo*11 gene, a meiosis-specific topoisomerase essential for meiotic recombination, and this observation was used as an explanation for the infrequent occurrence of the sexual cycle of *B. bassiana* [12]. The *spo*11 gene was however present in the genomes of all five *B. bassiana* isolates analysed in this study, and the conclusion about its absence in *B. bassiana* [12], based on a draft genome assembly, was thus erroneous.

### Isolate-specific genes from Bb8028 include candidate virulence genes

**Fig. 4:**
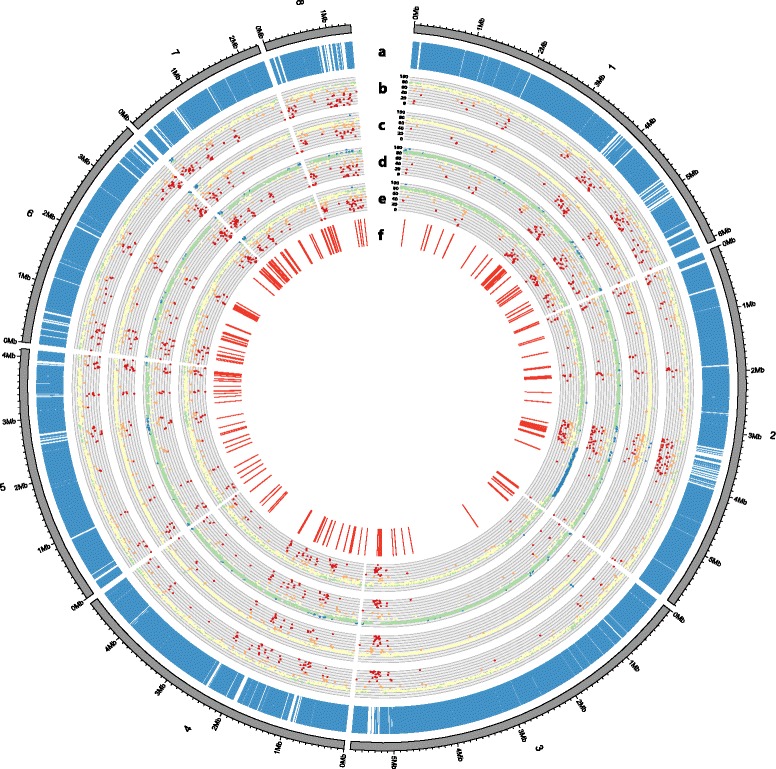
Circular visualization of the alignment of genome sequencing reads of four isolates of *Beauveria bassiana* with respect to the Bb8028 reference assembly. The eight largest contigs are numbered 1 to 8 along the perimeter of the outer circle. The tracks represent the location of predicted genes for Bb8028 (**a**); mean read coverage (per 10 kb) for isolates Bb1520 (**b**), Bb2597 (**c**), Bb4305 (**d**) and Bb5078 (**e**); and location of the unique genes for Bb8028 (**f**). In tracks (**b**) to (**e**), colours represent the percentage of coverage against the Bb8028 reference assembly: red: 0–45%, orange: 45–63%, yellow: 63–77%, green: 77–90%, and blue: 90–100%)

Isolate Bb8028 was previously characterized as the most virulent towards malaria mosquitoes [16]. Therefore, we hypothesised that the high virulence could be due to the presence of genes unique to Bb8028 that are related to fungal virulence. In order to detect regions uniquely present in Bb8028, all reads from the four other isolates were mapped to the genome of Bb8028 (parsed in bins of 10 kb in size) and regions with very low or no read coverage were detected (Fig. 4). A total sequence length of 0.71 Mb (2% of the genome of Bb8028) displayed very low coverage in the four other isolates, these regions encompassed 390 genes. To independently verify whether these genes were indeed unique, raw reads of the four other isolates were then mapped to individual genes of Bb8028. This approach identified 252 genes from Bb8028 with low or no coverage in all four other isolates. Comparison of the two datasets yielded 163 genes that were identified by both methods (Additional File 3), which we therefore considered to be unique to isolate Bb8028. The location of these Bb8028 genes on the eight largest contigs was analysed. Interestingly, the genes showed a tendency to cluster together, especially close to telomeres (most notably at the ends of contigs 3, 5 and 6, and at the beginning of contigs 7, 8 and 12; Additional File 3). The unique Bb8028 genes were functionally annotated to evaluate their potential role in pathogenicity. Of the 163 genes, 57 (35%) have homology to genes in the Pathogen Host Interaction Database (PHI-base) [53], 47 (25.2%) were hypothetical proteins, and 27 (16.6%) were predicted to contain signal peptides. Both the number of genes classified as hypothetical proteins and those predicted to be secreted were higher than expected when compared to the complete gene content (15%, *χ* ^2^ = 12.02, d.f. = 1, *p* = 0.000527; and 10.4%, *χ* ^2^ = 5.77, d.f. = 1, *p* = 0.01629, respectively). By contrast, the number of genes homologous to genes represented in PHI-base follows the expectation based on the whole gene set (29.4%, *χ* ^2^ = 2.1223, d.f. = 1, *p* = 0.1452). An overrepresentation of gene ontology terms was not detected in the 163 genes that are unique to Bb8028.

Secondary metabolites play a role in fitness and virulence of fungal (entomo)pathogens [54]. A Non-Ribosomal Peptide Synthetase (NRPS) gene cluster is present in the unique gene set of Bb8028 at the start of contig 8. The NRPS gene (BB8028_0008g00080) has homology (52–54%) to several *Metarhizium* species, with the difference that BB8028_0008g00080 has a single adenylation domain and is 1059 amino acids in length, while its *Metarhizium* homologs possess two adenylation domains and encode proteins almost double in size. In addition, Bb8028 contains a unique polyketide synthase gene (BB8028_0001g06000.1).

Five genes unique to Bb8028 (thus lacking in the four other *B. bassiana* genomes) encode proteins possessing bacterial enterotoxin-like domains. Four of these contain a heat-labile enterotoxin domain (IPR001144) while one protein displays 54% similarity to the *Vibrio cholera* enterotoxin alpha chain domain (IPR008992). Outside the kingdom Eubacteria, these enterotoxin genes have homology almost exclusively to *Beauveria, Cordyceps* and *Metarhizium* species, all of which are entomopathogenic fungi (data not shown). Bb8028 also possesses 11 peptidase genes (from various types) that have no orthologs in any of the other four *B. bassiana* isolates. Furthermore, two genes encoding the enzyme cyanamide hydratase are uniquely present in Bb8028. Cyanamide hydratase converts cyanamide into urea, and has been studied in relation to fungal survival in the soil since cyanamide is used as a fertilizer and is also synthesized by vetch species [55, 56]. Bb8028 also contains 13 unique genes involved in transport processes, of which five belong to the major facilitator (MFS) superfamily, four to the family of ATP-binding cassette (ABC) transporters, and the remaining four are dedicated siderophore and dicarboxylate transporters, as well as a sodium/proton antiporter. ABC transporters can be involved in (multidrug) resistance to fungicides, as well as the secretion of antibiotics and toxins [57]. Recently, five ABC transporters were characterized in *B. bassiana* and found to be implicated in fungicide resistance, oxidative stress tolerance, and in virulence [58].

Finally, among the gene set unique to Bb8028 are also genes encoding enzymes putatively involved in the modification of chromatin proteins, which may modulate changes in gene expression [59]. Specifically, these genes encode three arginine deiminases and a putative histone arginine N-methyltransferase.

Although a detailed analysis of differential gene expression could not be done with the RNA data available due to the lack of sample replication, an exploratory examination was done to see whether unique genes of Bb8028 were highly expressed. Specifically, we looked for genes that had at least (1) double the expression (in Fragments Per Kilobase of transcript per Million mapped reads (FPKM)) in the library isolated from infected mosquitoes compared against the library from fruiting bodies or mycelia, and, (2) a value of at least one FPKM. Of the 163 genes, 15 genes fulfilled these criteria. Notably, three of the five genes annotated as a toxin were among these 15 genes (Additional File 4). Also one of the arginine deiminase genes and one of the cyanamide hydratase genes displayed a high expression in the mosquito library as compared to the other two libraries.

### No clear pattern of positive selection in genes of Bb8028

Genes with a signature of positive selection may contribute to the adaptation to a changing environment and could be related to virulence. Therefore, we investigated whether genes were under positive selection in Bb8028. Ka/Ks ratios were calculated in pairwise comparisons between orthologs in Bb8028 and each of the four other isolates, separately. We detected 10, 5, 11, 10 genes of Bb8028 with Ka/Ks > 1 when compared to their orthologs in Bb1520, Bb2597, Bb4305, Bb5078, respectively. In total, 18 genes are considered to be under selection in Bb8028, but intriguingly no gene was shared among the four pairwise comparisons (Additional File 5). Among the genes under positive selection are two calmodulin-dependent protein kinase genes and an ABC transporter gene, while most of the other genes were of unknown function.

## Discussion

In this study, we provide and compare the genomes of five isolates of *B. bassiana* that were previously characterized in a large-scale analysis of virulence against the malaria mosquito (*An. coluzzii*) [16]. These isolates have different levels of virulence, with Bb8028 being the most virulent isolate, followed by Bb2597, Bb1520, Bb4305, and finally Bb5078. Comparative genomics at the intraspecific level is an approach to determine the genetic basis of phenotypic variations detected between different isolates. The genome of *B. bassiana* was previously compared to other entomopathogenic fungi [12], and in order to avoid a duplication of this previous work, we focused on comparison between *B. bassiana* isolates, with an emphasis on Bb8028. A pan-genome analysis indicated that the core genome of *B. bassiana* consists of 7,341 orthologous gene clusters, while the pan-genome consists of 13,068 orthologous gene clusters. These numbers suggest that *B. bassiana* has an open pan-genome, as may be expected for a species that can typically colonize multiple environments [51].

The isolates analysed have a high level of diversity with 467,292 SNPs between Bb4305 and Bb8028, and 87,119 SNPs between Bb5078 and Bb8028. Previous studies have suggested that *B. bassiana* is not monophyletic, but rather is composed of several clusters that are morphologically identical [48, 60, 61]. One study [61] described seven phylogenetic clusters in *B. bassiana* that consisted of isolates which did not show any geographic distribution but had common climate characteristics, while a different study [48] described isolates that were distributed in five clusters in a particular hedgerow habitat, however, genetic distances were not determined on a genome-wide scale in these studies. A comparative genomic study of seven isolates of the plant pathogenic fungus *Penicillium expansum* revealed a genetic diversity between 1.3 and 7.1 SNPs/kb [62], while between two isolates of *Aspergillus niger* it was 7.8 SNPs/kb [63]. In both these cases, the genetic diversity was lower than observed in this study (2.9–16.8 SNPs/kb). The finding that *B. bassian*a possesses a high genetic diversity seems to be in agreement with the earlier suggestions that *B. bassiana* is not monophyletic [48, 60, 61] and warrants further studies to elucidate the species phylogeny on a whole genome scale.

*B. bassiana* normally reproduces asexually, and for a long time it was believed to be exclusively clonal. Developmental and phylogenetic studies, however, have linked it to the teleomorph *Cordyceps bassiana* [64, 65]. In the five isolates studied, the *MAT*1-1 and *MAT*1-2 loci were identified based on homology to the typical fungal mating type genes, encoding the alpha domain protein and the high mobility group box domain, respectively. The presence of either of two distinct alleles of the *MAT* locus in each *B. bassiana* isolate indicates that the species is putatively heterothallic. A new *MAT* gene was detected next to the *MAT*1–2–1 gene which, based on homology to a newly described gene in *Villosiclava virens* [52], is referred to as *MAT*1–2–8 following the nomenclature proposed by Dyer et al. [66]. We attempted to sexually cross different isolates of *B. bassiana* in several combinations to generate fertile fruiting bodies. Based on the mating type, six combinations were possible (Additional File 6). Unfortunately, none of the crosses generated ascospores, but the cross between Bb8028 and Bb5078 generated fruiting body-like structures without ascospores; these structures were used to generate a transcriptome library. When taking into consideration the phylogenetic clusters found, we realized that only Bb8028 and B5078 belong to the same cluster and had the lowest number of SNPs/kb, compared to the other possible combinations. Since *B. bassiana* could be polyphyletic, and reports have found a single mating type in several populations [48], it is not surprising that *B. bassiana* is limited mainly to asexual reproduction. It is worthwhile to invest in a reproducible protocol for sexual crosses under laboratory conditions. The ability to achieve sexual reproduction will enable genome-wide association studies (GWAS) of quantitative virulence in the offspring of two isolates that markedly differ in virulence, for instance Bb8028 and Bb5078.

In this analysis, we identified a suite of genes that are unique to Bb8028. Among these are a PKS gene and a gene cluster comprising an NRPS gene with homology to a *M. anisopliae* gene annotated as tyrocidine synthase (KJK81377). Tyrocidine has antibiotic activity, and the peptide synthesized by the NRPS gene product could thus have antibiotic activity. The set of genes unique to Bb8028 also comprised five genes encoding bacterial-like toxins and 13 transporter genes. The *B. bassiana* genome contains many more bacterial-like toxins than most other fungi [12]. Interestingly, we found preliminary evidence that some of the bacterial-like toxins are highly expressed in fungal infection of mosquitoes. A recent study [67] analysed gene expression in *B. bassiana* ARSEF 2860 infecting *Plutella xylostella* larvae at three different time points after inoculation (24 h, 36 h, 48 h). In the latter study, an enrichment of genes with functions in antioxidant activity, peroxidase activity, and proteolysis was detected among the genes that are highly expressed in *B. bassiana* during infection of the host insect. In contrast, toxin genes were not reported to be highly expressed during infection of *P. xylostella* larvae [67]. The mortality rate of *P. xylostella* larvae infected by *B. bassiana* at 48 h was 33%, which is much lower than the mortality rate of almost 100% of infected mosquitoes at the time that we sampled RNA for library construction. The infection cycle of *B. bassiana* is a complex process which includes several developmental transitions that require an intricate genetic regulation [15]. This biological complexity may explain why no overlap is found between these studies. Furthermore, *B. bassiana* is capable of infecting a wide range of hosts that have different chemical and physical compositions, which most likely requires a tailored response for each host-pathogen interactions.

## Conclusions

The availability of a near-complete genome assembly of Bb8028 provided with a manually curated gene set, adds significant value to the previously published draft genome of *B. bassiana* [12]*.* As argued by [68], a high quality assembly including proper assembly of centromeres, telomeres, and other repetitive elements provides the ability to characterize structural rearrangements and facilitates a better identification of secondary metabolite gene clusters. Therefore, a high quality genome is a key step to uncover the biological complexity of an organism, as our analyses showed. The Bb8028 genome assembly represents a significant resource for future research into the biology of this entomopathogenic fungus and its relevance as a biological control agent. Understanding the key factors involved in virulence of *B. bassiana* will improve our methods to use this fungus as an effective and sustainable biological control agent against, amongst others, vectors of human diseases.

## Additional files

Additional file 1: Summary of the genome quality benchmarks performed with BUSCO. (XLSX 10 kb)
Additional file 2: PCR verification of the presence of MAT1–1–1 (odd numbers) or MAT1–2–1 (even numbers) in the genomes of different isolates of *Beauveria bassiana*. (1–2) Bb1520, (3–4) Bb2597, (5–6) Bb4305, (7–8) Bb5078 and (9–10) Bb8028. (JPG 62 kb)
Additional file 3: Summary of the unique genes of Bb8028. (XLSX 23 kb)
Additional file 4: Summary of the unique genes of Bb8028 that were highly expressed in the infected mosquito library. (XLSX 11 kb)
Additional file 5: Summary of genes of Bb8028 that under positive selection. (XLSX 10 kb)
Additional file 6: Additional_file_6.xlsx. Fruiting body formation from isolates of *Beauveria bassiana* with different mating types. (XLSX 9 kb)
