# Supplementary figures and images for: Comparative genomics of Beauveria bassiana: uncovering signatures of virulence against mosquitoes
Source: BMC Genomics. 2016 Dec 1;17:986. doi: 10.1186/s12864-016-3339-1 (PMC5134283; doi:10.1186/s12864-016-3339-1)

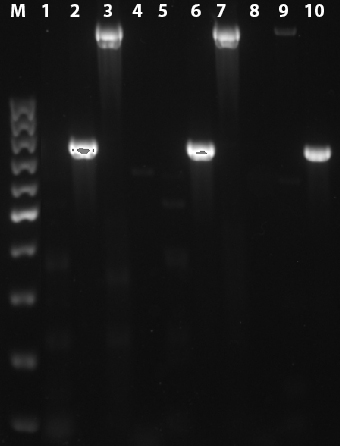

Supplement: Additional file 2: — PCR verification of the presence of MAT1–1–1 (odd numbers) or MAT1–2–1 (even numbers) in the genomes of different isolates of Beauveria bassiana. (1–2) Bb1520, (3–4) Bb2597, (5–6) Bb4305, (7–8) Bb5078 and (9–10) Bb8028. (JPG 62 kb) [file 12864_2016_3339_MOESM2_ESM.jpg]
